# Thalamic Volume Is Reduced in Cervical and Laryngeal Dystonias

**DOI:** 10.1371/journal.pone.0155302

**Published:** 2016-05-12

**Authors:** Jeff L. Waugh, John K. Kuster, Jacob M. Levenstein, Nikos Makris, Trisha J. Multhaupt-Buell, Lewis R. Sudarsky, Hans C. Breiter, Nutan Sharma, Anne J. Blood

**Affiliations:** 1 Mood and Motor Control Laboratory, Massachusetts General Hospital, Charlestown, MA, United States of America; 2 Laboratory of Neuroimaging and Genetics, Massachusetts General Hospital, Charlestown, MA, United States of America; 3 Center for Morphometric Analysis, Massachusetts General Hospital, Charlestown, MA, United States of America; 4 Department of Neurology, Massachusetts General Hospital, Boston, MA, United States of America; 5 Department of Psychiatry, Massachusetts General Hospital, Boston, MA, United States of America; 6 Division of Child Neurology, Boston Children’s Hospital, Boston, MA, United States of America; 7 Department of Neurology, Brigham and Women’s Hospital, Boston MA, United States of America; 8 Psychiatry Neuroimaging Laboratory, Brigham and Women’s Hospital, Boston, MA, United States of America; 9 Harvard Medical School, Boston, MA, United States of America; 10 Warren Wright Adolescent Center, Department of Psychiatry and Behavioral Sciences, Northwestern University Feinberg School of Medicine, Chicago, IL, United States of America; 11 Athinoula A. Martinos Center for Biomedical Imaging, MGH, Charlestown, MA, United States of America; UCLA, UNITED STATES

## Abstract

**Background:**

Dystonia, a debilitating movement disorder characterized by abnormal fixed positions and/or twisting postures, is associated with dysfunction of motor control networks. While gross brain lesions can produce secondary dystonias, advanced neuroimaging techniques have been required to identify network abnormalities in primary dystonias. Prior neuroimaging studies have provided valuable insights into the pathophysiology of dystonia, but few directly assessed the gross volume of motor control regions, and to our knowledge, none identified abnormalities common to multiple types of idiopathic focal dystonia.

**Methods:**

We used two gross volumetric segmentation techniques and one voxelwise volumetric technique (voxel based morphometry, VBM) to compare regional volume between matched healthy controls and patients with idiopathic primary focal dystonia (cervical, n = 17, laryngeal, n = 7). We used (1) automated gross volume measures of eight motor control regions using the FreeSurfer analysis package; (2) blinded, anatomist-supervised manual segmentation of the whole thalamus (also gross volume); and (3) voxel based morphometry, which measures local T1-weighted signal intensity and estimates gray matter density or volume at the level of single voxels, for both whole-brain and thalamus.

**Results:**

Using both automated and manual gross volumetry, we found a significant volume decrease only in the thalamus in two focal dystonias. Decreases in whole-thalamic volume were independent of head and brain size, laterality of symptoms, and duration. VBM measures did not differ between dystonia and control groups in any motor control region.

**Conclusions:**

Reduced thalamic gross volume, detected in two independent analyses, suggests a common anatomical abnormality in cervical dystonia and spasmodic dysphonia. Defining the structural underpinnings of dystonia may require such complementary approaches.

## Introduction

Dystonia is a neurologic disorder characterized by involuntary, abnormal twisting or deforming movements that lead to painful and debilitating positions and/or postures. Although a prerequisite for the diagnosis of primary dystonia is an absence of pathology on clinical imaging, neuroimaging research has demonstrated numerous subtle structural and/or connectivity abnormalities in dystonia, using measures such as voxel based morphometry (VBM) and diffusion tensor imaging (DTI).[1, 2] Dystonia patients and their unaffected relatives exhibit some overlapping neuroimaging abnormalities,[3–5] suggesting that heritable factors that induce structural changes in motor control regions may predispose individuals to dystonia. Prior morphometry studies that evaluated multiple types of dystonia identified type-specific grey matter abnormalities, but many of these abnormalities were not consistent between and within types.[1, 2, 6, 7] In contrast, common abnormalities across forms of dystonia have been identified[3, 6] raising the prospect that, while dystonia appears to be a circuit or systems level disorder,[8, 9] identifying abnormalities shared across dystonia types may give clues to focal origins of dystonia pathophysiology.

There is little literature on gross volumetric measures (i.e., direct segmentation and counting of voxels within a region) in dystonia. Black et al. measured gross volume using paired stereology and manual region-tracing techniques in patients with blepharospasm or hand dystonia, showing increased putaminal volume in dystonia patients.[10] Vasques et al. utilized manual anatomic labeling followed by automated putaminal volume determination in patients with generalized dystonia, demonstrating that reduced gross volume in the putamen correlates with reduced response to deep brain stimulation (DBS).[11] Similar gross volumetric assessments have been used to study secondary and heredodegenerative dystonias as well, following thalamic stroke[12] and in Machado-Joseph disease.[13] To our knowledge, these gross volumetry methods have not been utilized to study a range of motor-control regions in cervical dystonia or spasmodic dysphonia.

Gross volume measures are complementary to the local, voxelwise tissue density and volume measures made by VBM, but the two measures are not interchangeable. As noted by Friston and Ashburner, “VBM is not a surrogate for classical volumetric analysis of well-defined anatomical structures.”[14] VBM measures of grey matter density can be converted into a volume-like measure, although this is indirect (it must be extrapolated from other VBM measures, each susceptible to distortion by group registration and smoothing).[15–17] Gross volumetry and VBM assess neuroanatomy through distinct approaches and at very different scales; while each technique may aid in the interpretation of the other, the two techniques do not “compete” for anatomic truth.[18] There have been a number of previous VBM studies in dystonia, detecting abnormalities across a range of sensorimotor regions,[1–7, 19–21] consistent with the “network”[22–24] or “systems level”[9, 25] aspect of dystonia.

Based on their different approaches to evaluating volume, gross volumetry and VBM changes in disease may not always change in parallel. Characteristics of local tissue volume, as measured by VBM, may in some cases influence gross volume measures. For example, a decrease in tissue density spanning many voxels within a brain region may lead to reduced gross volume of that region. However, local and global volume changes may also occur independent of one another and thus may be detected by one volumetric technique but not the other. For example, changes in cellular populations or morphologies may alter tissue density without changing overall volume of a region or, conversely, gross volume may differ across groups with no differences in local density. Following this logic, distinct pathophysiologies of disease likely produce differential effects on local versus gross volumetry. Indeed this is supported by the literature; gross volumetry and VBM methods show differential sensitivity to structural changes in developmental conditions, such as dyslexia[26] and autism,[27] as well as in normal aging.[28] Therefore, using gross volumetric measures in parallel with local volume assessment (through VBM) may be critical to gaining a complete picture of brain volume measures in dystonia.

In the current study, we evaluated gross volume of eight sensorimotor regions reported in previous dystonia studies,[1, 3, 6, 21] across two forms of primary focal dystonia, where subjects with dystonia were matched one-to-one with healthy controls. The *a priori* regions included: caudate, putamen, globus pallidus, Brodmann area 6 [premotor and supplementary motor areas], pre- and postcentral gyrus, cerebellar cortex, and thalamus. Analysis followed three general steps. First, we used automated segmentation to make gross volume measurements in each subject, followed by patient vs. control comparison of summed Left and Right volumes in *a priori* regions. Second, for all regions in which this automated analysis yielded significant group differences, we implemented a manual segmentation method based on anatomic landmarks with blinded anatomist oversight to confirm these findings. Third, because of the abundance of VBM studies in dystonia, we performed VBM as a complementary measure and a reference point for the current study relative to others in the dystonia literature. We conducted VBM of the whole brain, and conducted independent VBM assessment restricted to any *a priori* regions for which gross volumetric differences were observed in the first two steps. As a final step, any *a priori* region with significant results in at least two of our three morphometric methods was correlated with clinical indices to facilitate interpretation.

## Methods

### Subjects

**Fig 1:**
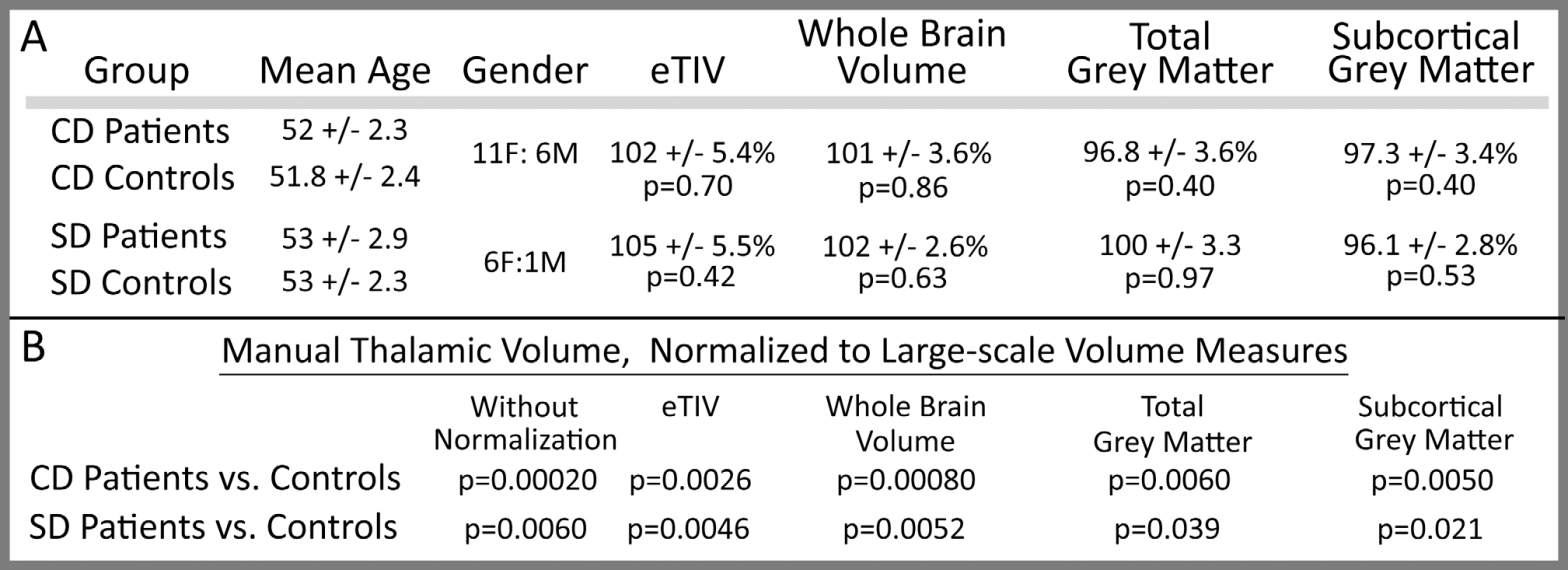
Demographic information and gross volumetric negative-control contrasts. Demographic and volumetric negative-control measures for each experimental group demonstrate high group similarity. Within each experimental group (cervical dystonia–CD; spasmodic dysphonia–SD), controls were matched to patients for gender, handedness, and age +/- five years (A). Each volumetric negative-control measure is expressed as the percentage of mean control volume (*e*.*g*., estimated total intracranial volume (eTIV) for CD patients is 2% larger for patients than for matched controls). No large-scale volumetric measures differed between patients and controls (A, p-values uncorrected). The reduction of thalamic volume in patients maintained significance following normalization for all volumetric negative-control measures (B).

Seventeen patients with idiopathic cervical dystonia (CD), negative for the DYT1 mutation, and seventeen healthy controls were matched one-to-one (as paired, demographically matched dyads) for age (+/- five years, Fig 1A), gender, and handedness. Seven patients with idiopathic spasmodic dysphonia (laryngeal dystonia; SD) who were negative for both DYT1 and DYT6 mutations, and seven healthy controls were matched one-to-one (as paired, demographically matched dyads) for age (+/- five years, Fig 1), gender, and handedness. The mean ages for patient and control groups were nearly identical, namely: CD patients: 52 ± 2.3 years, CD controls: 51.8 ± 2.4 years; SD patients: 53 ± 2.9 years, SD controls: 53 ± 2.3 years.

Minimal differences in age between each patient:control dyad (mean difference: 1.88 years) had no effect on volumetric measures; multiple linear regression of (A) subject age or (B) age mismatch within each dyad yielded non-significant p-values for both CD (A, p = 0.52; B, p = 0.56) and for SD (A, p = 0.83; B, p = 0.94) demonstrating that age within a patient:control dyad was well matched. Handedness was determined using the original Edinburgh Handedness Inventory;[29] one CD patient and matched control were left handed, and all other CD and SD patients and controls were right handed. Twelve subjects were described previously,[30] though none were assessed by volumetry. Six SD controls were drawn from the pool of CD controls; one SD control was independent of the CD cohort. All controls for SD were matched as described for CD. This cohort is similar in size to prior dystonia VBM studies.[3–7, 19] For patients treated with botulinum toxin (BTX) injections, scanning was conducted at the end of a treatment period (within a week before next scheduled injection) so that effects of BTX had waned and symptoms were maximal. Fourteen control subjects were scanned twice, with four to five weeks between scans. This group allowed us to estimate the inter-scan variability for automated volumetry. All participants were provided with printed materials describing the research protocol and were encouraged to ask questions regarding the study. We obtained written consent for all participants. The human studies section of the Institutional Review Board for Partners HealthCare System approved this protocol, including our consent procedure. All research was conducted in accordance with the principles in the Declaration of Helsinki.

### Clinical Variables

Age at symptom onset and symptom duration was derived from retrospective patient estimation. For CD, the Tsui score, a validated measure of CD severity,[31] was determined on day of scanning. For SD, the voice-related quality of life score (V-RQOL) was assessed on the day of scanning.[32] For subjects who received BTX injections within three months of scan day (15/17 CD patients), the site and amount of BTX injection were obtained from clinic notes. The laterality of symptoms was assessed using the ratio of BTX units injected into left versus right-sided muscles. This “hemispheric BTX ratio” was calculated as follows: (units injected into left-sided muscles minus units injected into right-sided muscles) / total units. The sternocleidomastoid produces rotation toward the contralateral side, counter to the direction of movement produced by other ipsilateral cervical muscles. Thus, we calculated the hemispheric BTX ratio in two ways–with the sternocleidomastoid counted ipsilateral (anatomic side) or contralateral (side of movement). The relationship between hemispheric BTX ratio and thalamic volume ratio did not differ substantially between these two calculation methods; the illustrated hemispheric BTX ratio utilized the “side of movement” calculation. Only 3/7 SD patients were treated with BTX, so the SD cohort were not assessed for BTX-related metrics.

### MRI Imaging

Subjects were scanned at the MGH Athinoula A. Martinos Center for Biomedical Imaging (Charlestown, MA). Thirteen CD patients and their thirteen one-to-one matched controls (each matched to a single patient for age, gender, and handedness), as well as seven SD patients and seven one-to-one matched controls, were scanned on a 3.0 Tesla Siemens Tim Trio MRI (Siemens AG, Medical Solutions, Erlangen, Germany); four CD patients and their four one-to-one matched controls (each matched to a single patient for age, gender, and handedness) were scanned on a Siemens 3.0 Tesla Allegra MRI, as published.[33] As matched patient-control dyads were always scanned in the same magnet, this change in magnet did not increase the likelihood of type I errors (false positives) for simple group comparisons. As matching was done separately for each magnet, any magnet-by-demographic or magnet-by-software interactions relating to group differences were minimized by the experimental design. However, given a potential increase in variance in patient/control dyad differences across scanners, inclusion of data from the two scanners had the potential to affect the likelihood of type II (false negative) errors. In addition, if mean age were to differ across scanners, observed age-related effects might be influenced by a differential ability of each scanner to detect these age-related differences. While the mean age of participants between the Allegra and Trio scanners was very similar (49.8 and 52.4 years, respectively), and thus unlikely to have an effect on results, this remained a potential caveat in interpreting age-related effects in our groups. Furthermore, given the more narrow age range and small size of the SD cohort, changes across the age span in the SD cohort are difficult to interpret, and may indeed be more likely related to either unmeasured clinical factors or sporadic variation.

During each subject’s scan session, we acquired two sagittal 3D Magnetization Prepared Rapid Gradient Echo (MP-RAGE) T1-weighted sequences, with both scans incorporated into subsequent automated segmentation: TR = 2730ms, TE = 3.31ms, TI = 1,000ms, flip angle = 7°, bandwidth = 195Hz/pixel, FOV = 256×256mm^2^, sampling matrix = 256×192pixels, 128 contiguous 1.33mm slices.

### Morphometric Analyses

#### Automated Segmentation

Automated segmentations of motor-control regions identified by prior structural dystonia studies [1, 3, 6] (listed in Introduction) were generated using *recon-all* (Freesurfer), a command that performs regional segmentation and measures gross regional volume in a conformed space (256×256×256 matrix, with coronal reslicing to 1mm^3^ voxels). All subjects were processed using Freesurfer version 5.1.0 using a single Linux workstation as suggested by Gronenschild et al.[34] The agreement between manual segmentation and automated volume determination under version 5.1.0 is significantly improved relative to earlier versions of Freesurfer.[35] As negative controls for regional segmentations, *recon-all* generated gross brain volume measurements for larger-scale regions (i.e., total number of voxels per region): total grey matter, subcortical grey matter, brain mask volume (BrainMaskVol, the residual volume following skull stripping), and estimated total intracranial volume (eTIV, the Freesurfer approximation of total intracranial volume, extrapolated from the transform of each brain from native to standard space). Descriptions of how the above measures are computed can be found at www.freesurfer.net/fswiki/eTIV and www.freesurfer.net/fswiki/BrainVolume. These control measures were assessed for differences between groups, and these control measures were also used to calculate normalized values for automated and manual segmentations (described below), correcting for individual differences in head size, cerebral size, and grey matter volume (normalization = [thalamic volume]/[negative control volume]). Prior investigators have noted the difficulty in determining the best method for normalizing regional volumes, including the potential for correction by ICV or other gross brain volume measures to minimize true group differences in volume.[36, 37]; therefore, we reported both raw, uncorrected volume and corrected regional volume (through multiple gross brain volume normalization approaches) to assess the consistency of findings across methods.

#### Manual Segmentation

Manual gross volume measures were performed in brain regions observed to be significantly different by automated gross volume assessment (*i*.*e*., thalamus). For these manual segmentations, we used the same MP-RAGE images used for automated segmentations. Manual segmentation followed precise, neuroanatomist-defined landmarks in the atlases of Talairach and Tournoux[38] and Mai *et al*.[39] to produce an anatomically correct segmentation. These segmentations were performed using the FreeView utility (Freesurfer). The resolution of 3 Tesla MRI is insufficient to quantitatively and reliably segment discrete thalamic nuclei; therefore, all thalamic subnuclei (the whole thalamus) were included. Direct measurement of gross thalamic volume for each subject was generated within FreeView. Segmentation landmarks were: Anterior: foramen of Monro; Superior: lateral ventricle and stria terminalis; Lateral (anterior): posterior limb, internal capsule, inclusive of grey matter islands; Lateral (posterior): fornix; ventral hypothalamic fissure in anterior sections, extending ventrolaterally to include lateral geniculate at the first section including the posterior commissure; Medial: 3^rd^ ventricle, including massa intermedia where present; Posterior: lateral ventricle. Manual segmentation of the whole thalamus was performed by one author, blinded to individual measures from the automated segmentation (JW). Manual segmentations were then evaluated and revised by senior authors AB and NM (blinded to both group and diagnosis). NM is a recognized neuroanatomist with two decades of anatomy research experience.

#### Voxel-based Morphometry

Automated assessment of gray matter volume per voxel was performed using previously-validated voxel-based morphometry (VBM) procedures.[40] In brief, T1 structural images were analyzed with FSL-VBM,[41] an optimized VBM protocol[42] utilizing FSL tools.[43] T1 structural images were skull-stripped and grey matter was segmented, then non-linearly registered to the MNI152 template. Resulting images were averaged and mirrored along the x-axis. All native-space grey matter images were non-linearly registered to separate dystonia type-specific templates for each of the patient/control cohorts (34 CD subjects for the CD template; 14 SD subjects for the SD template) and corrected for local expansion or contraction (i.e., modulation step). The modulated grey matter images were then smoothed with an isotropic Gaussian kernel with sigma = 3mm. Voxelwise volume was then extrapolated from the Jacobian warp field produced during non-linear registration. In addition to whole-brain comparisons, extraction and comparison of only the thalamic voxels was carried out using a composite thalamic mask. This mask was produced by manually segmenting the thalamus from the average template-registered grey matter image produced by FSL-VBM. Finally, to more closely parallel the non-voxelwise approach of gross volumetry, we used a region of interest (ROI)-based approach and extracted the average VBM measures across all voxels within the thalamic mask for each subject and compared across dystonia and control cohorts.

### Statistical assessment

All statistical tests utilized Stata (College Station, TX, v13), except for the ANCOVA and ANOVA tests, which were run using SPSS.

#### Automated and Manual Segmentation Comparisons

Individual gross volume measures for bilateral motor-control regions (summed Right + Left hemisphere), large-scale negative-control areas, and manual thalamic segmentations (both native space and normalized by negative-control volumes) were compared between patients and controls for each disease (CD or SD). For both automated and manual thalamic gross volume measures, we conducted these comparisons using paired, two-tailed Student’s t-tests. The paired approach increased our ability to remove any potential variance across each population relating to age, gender, and/or scanner. The requirements for paired t-tests (normal distribution, equal variance, and one-to-one group matching criteria) were evaluated and verified.[44–46] P-values for gross volume were Bonferroni corrected for multiple comparisons (eight *a priori* regions, hence significance threshold = 0.05 ÷ 8 = 0.00625). Gross volumetric negative control analyses are reported with the significance threshold uncorrected (i.e, p = 0.05) for the most conservative approach. Thalamic volume corrected for large-scale negative-control areas (e.g., intracranial volume) did not undergo subsequent Bonferroni correction; hence, p-values were not double-corrected.

#### Voxel-based Morphometry Comparisons

Voxel-wise permutation-based non-paired, non-parametric testing was applied to compare dystonia and control cohorts using the FSL tool *randomise*, utilizing threshold-free cluster enhancement (TFCE) and 5000 permutations per voxel. CD patients were compared with CD controls; SD patients were compared with SD controls. *Randomise* comparisons were made for both whole-brain and thalamus-specific voxels. To compare the gross volume measures derived from direct segmentations with the voxelwise measures derived from VBM, we assessed groupwise differences in mean VBM signal for all thalamic voxels in a paired, two-tailed t-test, with a significance threshold of p<0.05.

#### Association of Gross Thalamic Volume with Clinical Variables

Assessing covariates of interest: We used a multiple linear regression to evaluate manually segmented thalamic volumes in relation to age at scan, age at symptom onset, duration of symptoms, dystonia severity, and BTX dose (total units injected, CD only). Before running the regression we assessed for collinearity between any of the covariates of interest. In the CD cohort, age of onset correlated with three other variables, including age at scan (R = 0.56; p = 0.019), duration of symptoms (R = 0.74, p = 0.0007), and BTX dose (R = 0.53, p = 0.029). No other covariates showed collinearity. Since multicollinearity is an exclusion criterion for the multiple regression, we excluded age of onset from the regression. Furthermore, since age at scan covaried with volumetry in patients and appeared to decline with age similarly in CD controls, any findings relating to age of onset would likely be explained by its collinearity with age at scan; therefore, we did not evaluate age of onset further in our CD cohort. For the SD cohort, duration of symptoms correlated with age at scan (R = 0.87, p = 0.010) and age of onset (R = 0.80, p = 0.030), so this factor was removed from our regression for this cohort. Correlation between the ratio of Left to Right hemisphere thalamic volume and the hemispheric BTX ratio was assessed by a separate linear regression.

Assessing potential interactions and removing covariates of no interest: In addition to evaluating associations with clinical variables, we also asked whether the broad age range in the CD cohort might have an influence on our group volumetric effects. Specifically, we evaluated patient versus control volume (in CD versus controls, as well as a separate analysis in SD versus controls) using an ANCOVA in which we controlled for age, using age as a regressor of no interest. Before running this analysis we evaluated the linearity of regressions and the homogeneity of regression slopes (i.e., to assess potential interactions of group and age) for each contrast to verify that the ANCOVA met these assumptions. The CD cohort met both assumptions, but the SD cohort did not meet the assumption of linearity; therefore, we did not run this analysis for the SD cohort. A simple ANOVA evaluating group by thalamic volume only (without removing the effects of age) was used in the CD cohort for comparison with the ANCOVA, to determine whether removal of age effects substantially changed the outcome. Finally, we used a complementary approach to evaluate group differences independent of age by assessing patient and control volume at the level of individual, age-matched patient/control pairs. Specifically, we compared single patients and single controls within a dyad (matched for age), and determined the number of dyads (for CD and for SD cohorts) in which patient volume was less than that of the matched control.

## Results

### Volumetry Measures

#### Gross Thalamic Volume by Automated and Manual Segmentations

**Fig 2:**
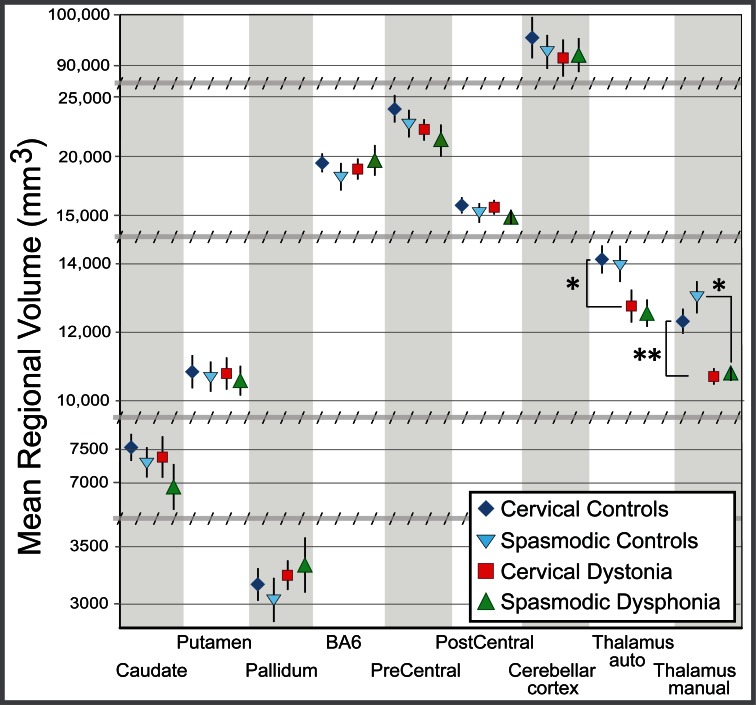
Regional automated and manual gross volume measures. A reduction in thalamic volume, not seen in other regions involved in the control of movement, was seen in both cervical dystonia and spasmodic dysphonia. Total volume (i.e., number of voxels in left plus right hemispheres) is shown for each region of interest (mean ± standard error of the mean). Given the large differences in volume between brain regions, the axis has been adjusted to focus on each cluster of values. Breaks in the y-axis are indicated by hashed horizontal bars. All p-values corrected for multiple comparisons (Bonferroni corrected significance threshold, p = 0.00625); * p≤0.0060; ** p = 0.00020. Abbreviation: BA6 = Brodmann Area 6; Thal auto = automated thalamic segmentation.

**Fig 3:**
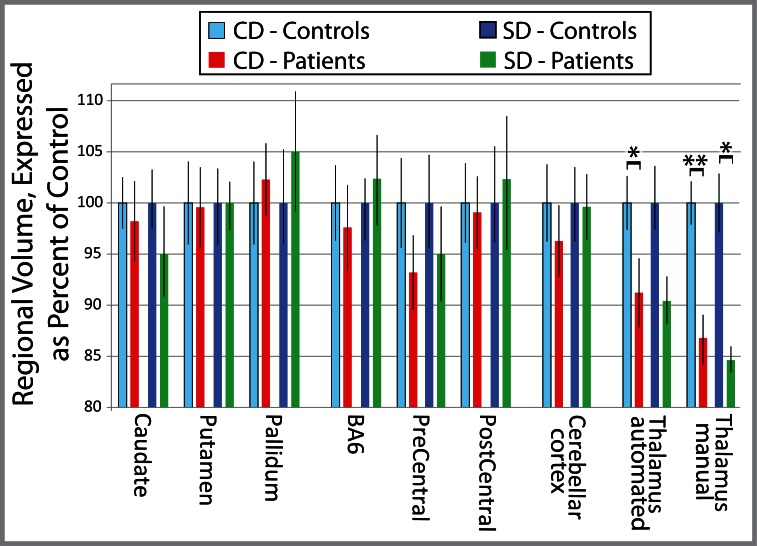
Relative regional gross volume measures in focal dystonia. Relative gross thalamic volume, normalized to matched controls, was less in dystonia patients. Total gross thalamic volume was normalized to the mean value for matched controls, expressed as percent difference ± standard error of the mean for each region of interest. This data is reformatted but otherwise identical to that in Fig 2. All p-values corrected for multiple comparisons, (Bonferroni corrected significance threshold, p = 0.00625); * p≤0.0060; ** p = 0.00020. Abbreviations: CD = cervical dystonia; SD = spasmodic dysphonia; BA6 = Brodmann Area 6.

A regionally-specific reduction in gross thalamic volume was observed in patients with CD or SD relative to matched healthy controls, with both automated (CD: 8.9% reduction, p = 0.0058; SD: 9.5% reduction, p = 0.16, NS) and manual (CD: 14% reduction, p = 0.00020; SD: 15% reduction, p≤0.0060) segmentations (Figs 1–3). None of the other seven *a priori* regions showed a significant difference from matched healthy controls by automated methods (all p>0.20). When normalized for intracranial volume or for whole-brain volume, the thalamus remained the only region significantly different between patients and controls (Fig 1, S1 Table). CD patients did not differ from SD patients in gross thalamic volume for either automated (p = 0.74) or manual (p = 0.78) segmentations. The mean change in automated thalamic volume measures between scans (14 controls, 4–5 weeks between scans) was 2.71% for uncorrected thalamic volume, and 2.32% for thalamic volume corrected for total intracranial volume.

Negative-control volumes did not differ between groups (Fig 1A), arguing that reduced thalamic volume in dystonia was not due to differences in head or brain size. Manual thalamic volume differences maintained significance when normalized to negative-control volumes (eTIV, brain mask volume, total grey matter, or subcortical grey matter, Fig 1B and S1 Table). Differences between left and right thalami were minimal (group means ranged from 1–2.5%) and non-significant, supporting use of summed volumes across hemispheres in this analysis.

#### VBM Measures

**Fig 4:**
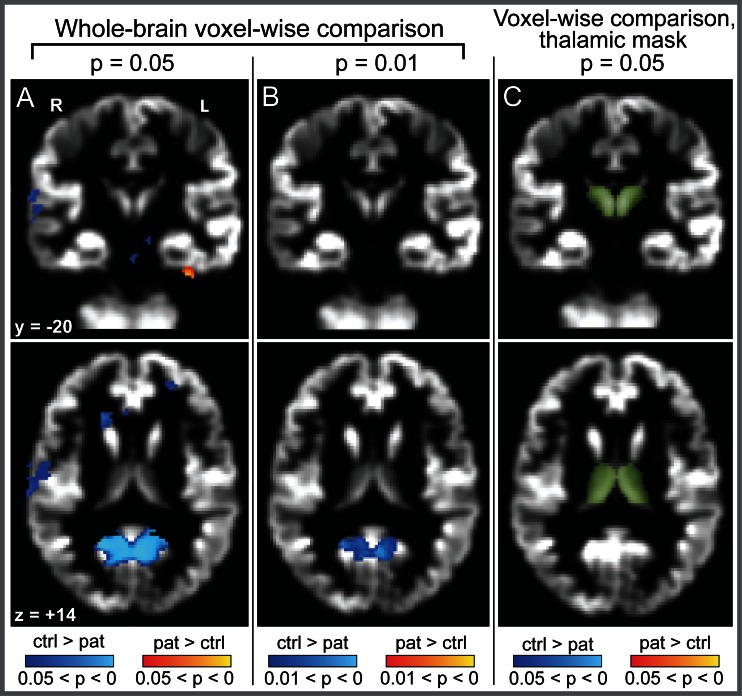
Voxel based morphometry in cervical dystonia. Voxel based morphometry demonstrated reduced gray matter local tissue volume in the posterior cingulate (A and B, blue voxels, shown at two significance thresholds, presented as family-wise uncorrected p-values), but no differences in the thalamus, in cervical dystonia (family-wise error corrected p = 0.9996). When the analysis was restricted to only those voxels in a thalamic mask (to minimize the loss of statistical power by multiple-comparisons correction; C, green voxels), no significant differences in local tissue volume were noted (p = 0.34). Significant voxels (A, B) and thalamic mask (C) overlie the mean gray matter structural image. Note that identical structural scans were used in VBM analyses and segmentation analyses (i.e., scans used in this figure were the same as those used for data in Figs 2, 3 and 5). VBM results were corrected using threshold-free cluster enhancement (TFCE). All axial and coronal views are from a single plane, indicated in MNI Talairach coordinates. Color bars at bottom indicate TFCE-corrected p-values for the images above. Abbreviations: pat = patients; ctrl = controls. R = Right hemisphere; L = Left hemisphere.

VBM detected no changes in voxelwise thalamic volume measures for either CD (Fig 4) or SD. This was true for whole-brain voxelwise comparison (most significant thalamic CD cluster, family-wise error corrected p = 0.9996, uncorrected p = 0.65, Fig 4A and 4B; most significant thalamic SD cluster, family-wise error corrected p = 0.967, uncorrected p = 0.19), for voxelwise comparison restricted to thalamic voxels, which optimizes the chances of detecting a regional change (lowest corrected p-value CD cluster, p = 0.34, Fig 4C; lowest corrected p-value SD cluster, p = 0.26), and for mean VBM difference across the thalamic mask, an ROI-based measure performed to parallel the ROI approach to gross volumetric assessment (p = 0.64 for CD; p = 0.32 for SD). For regions outside the thalamus, the VBM cluster of greatest significance was a reduction in local tissue volume in the posterior cingulate in CD patients (Fig 4A and 4B, p threshold = 0.01, cluster size = 452 voxels) that did not survive whole-brain correction for multiple comparisons (corrected p = 0.43). This uncorrected analysis is included solely to illustrate that the VBM method produced a meaningful result–the lack of significant VBM findings in the thalamus does not reflect a general failure of the method. VBM detected no significant voxelwise volume differences between SD patients and controls (most significant cluster, p = 0.184).

### Correlations between Gross Volumetric Measures and Clinical Indices

**Fig 5:**
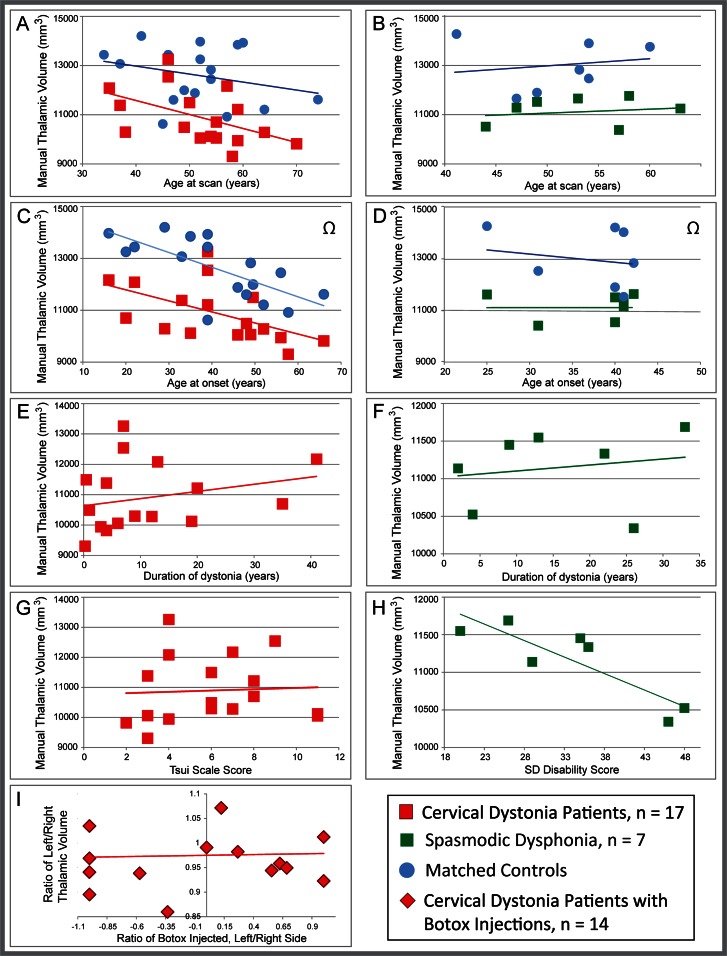
Relationship of clinical measures to gross thalamic volume in CD patients. The relationship between individual gross thalamic volume (manually segmented, in mm^3^) and clinical measures suggests that reduced gross thalamic volume is a risk factor for dystonia, and is not a secondary effect of dystonia symptoms. Data are shown for all variables, even if excluded from regression models, so raw data for CD and SD can be viewed and compared. Both CD patients and controls (A) showed declining volume with age. Patient:control gross thalamic volume showed a qualitative divergence with age between CD and controls, but the divergence was not statistically significant. (B) There was no relationship between volume and age in the SD cohort (for either SD or controls), presumably reflecting the smaller age range in this cohort. Likewise, there was no divergence of slopes with age between SD and controls. Age at CD onset (C) appeared to correlate with gross thalamic volume, but this was likely driven by the relationship between age at scan and age of onset; age at SD onset (D) was not correlated with volume. Gross thalamic volume did not correlate significantly with duration for either CD (E); this relationship was not evaluated statistically for SD due to collinearity with other variables, but the positive slope suggests no indication of a decline in volume with increasing duration (F). Thalamic volume also did not correlate with severity of dystonia for either CD or SD in the multiple regression model, as measured by the Tsui scale for CD (G) or the voice-related quality of life score for SD (H), although the SD relationship to severity showed a trend toward significance (V-RQOL, p = 0.056), and appeared significant when evaluated post hoc as a single variable (p = 0.012). The asymmetry of muscles affected with cervical dystonia (as gauged by laterality of units of botulinum toxin injected) did not correlate with asymmetries in thalamic volume (I, p = 0.89). Note that for (C) and (D), thalamic volumes for control subjects are plotted vs. the age at onset for the matched patient, as control subjects do not have an age at onset. Volume for control subjects is included in C and D as a reference only (designated by Ω), to illustrate that patient:control differences persist (and in fact are more robust) when demographics (including age) are matched: with the exception of a single CD/control dyad, every patient showed lower volume than his/her matched control.

Assessing covariates of interest: For CD patients, our multiple regression model showed that age at scan was associated with progressive reduction in thalamic volume (Fig 5A, p = 0.0073). Thalamic volume in CD showed no relationship with severity of dystonia (Fig 5E and 5G), while duration and total BTX dose trended towards significance as part of our four-variable model (p = 0.054 and p = 0.072, respectively). When evaluated as single, independent variables in relation to thalamic volume, however, neither duration nor BTX dose showed a trend or significance (R = 0.25, p = 0.33 and R = 0.042, p = 0.87, respectively). Hemispheric asymmetries in thalamic volume had no correlation with asymmetries in symptoms, as measured by the location and number of units of BTX used (Fig 5I). In contrast, thalamic volume in SD had no significant relationship with age at scan or age at onset (Fig 5D and 5F), while thalamic volume and severity trended toward significance as part of our three variable model (p = 0.056; Fig 5H). When evaluated post hoc as a single, independent variable, severity of SD correlated negatively with thalamic volume (R = -0.86, p = 0.012; Fig 5H). Despite exclusion of age of onset in CD and duration in SD in multiple regressions due to multicollinearity with other variables, we include scatterplots of all variables in relation to thalamic volume in Fig 5 for comparison of data across CD and SD, with the goal of fueling future hypotheses about these variables.

Assessing potential interactions and removing covariates of no interest: Both CD and control groups showed an age-related decline in volume (Fig 5A). ANCOVA testing showed that although the slope of the CD patients appeared steeper with age than controls, there was no significant interaction between group and age (F = 0.51, p = 0.479). Thus, it was valid to use the ANCOVA to remove effects of age in this cohort. This analysis demonstrated that thalamic volume differences between CD patients and controls remained and were slightly greater when the effect of age at scan was removed (Fig 5A; F = 21.348, p<0.0001, as compared with F = 19.385, p<0.0001 without age effects removed). This finding was consistent with the fact that all but one CD patient showed reduced volume relative to his/her demographically matched control (as illustrated by direct pairings in Fig 5C). Neither SD nor control groups showed age-related decline (Fig 5B); although the SD cohort did not meet this linearity assumption necessary to run the ANCOVA, we made a similar qualitative observation that all SD dyads showed patient < control volume, that is, group results were seen at the individual level when demographics were matched (as illustrated by direct pairings in Fig 5D).

## Discussion

In this study, both automated and manual segmentation approaches revealed a significant reduction in gross thalamic volume in two types of focal dystonia [cervical (CD) and laryngeal (SD)], a difference not found in other motor control regions. While some inter-scan variability in volume assessments is expected, the volume reduction identified in our automated segmentations (CD: 8.8%, SD: 9.4%) is three-fold larger than both the previously-reported inter-scan variability in Freesurfer’s thalamic volume determination[47] (2.96%) and our own inter-scan variability for control subjects using Freesurfer (2.71%). In contrast to these patient:control volumetric differences, CD and SD patient groups did not differ in thalamic volume. Reduction in gross thalamic volume in dystonia did not simply reflect a global loss of brain volume; global measures of volume did not differ between patients and controls, and thalamic volumes corrected for global volume measures maintained significance (Fig 1A and 1B).

Dysfunction in cortico-striato-pallido-thalamo-cortical loops has been postulated to underlie some forms of dystonia. Across multiple forms of focal dystonia, functional imaging studies have reported abnormal thalamic activation; while the majority of these studies identified increased thalamic function,[48–53] reductions have also been observed.[54] Functional and structural abnormalities in the thalamus may also reflect pathophysiologic changes in nuclei that are “upstream” in the cortico-thalamic motor control loops. Indeed, increased iron deposition in the globus pallidus, the primary output nucleus linking the striatum to the thalamus, was recently described in cervical dystonia.[55] Structural abnormalities of the thalamus and diverse basal ganglia nuclei have also been observed in primary dystonias using methods complementary to the ones used in the current study. Voxel based morphometry is the most commonly utilized structural research imaging tool used to study the focal dystonias. Results of these studies have been heterogeneous, both within and between dystonia types,[1, 2] possibly resulting from differences in details of the VBM algorithm, difficult-to-match demographic variables (such as duration and laterality), or true etiologic heterogeneity across dystonias.[6] In cervical dystonia, thalamic grey matter density has been reported to be increased,[6] decreased,[1] or unchanged.[7, 56] Similarly, in another focal dystonia, blepharospasm, grey matter density in the putamen has been reported to be increased,[19] decreased,[6] or unchanged[5], while thalamic grey matter density in blepharospasm has been found to be reduced.[6] Diffusion tensor imaging has detected reduced cerebello-thalamic connectivity in DYT1 mutation carriers,[57] and evidence for left/right asymmetries in cerebellothalamic connectivity in cervical dystonia.[30] The thalamus is also one of the most common sites implicated in post-stroke dystonia,[12, 58, 59] emphasizing its potential role in dystonia independent of pathogenesis.

Where do the current findings fit into this complex assemblage of abnormalities in focal and other dystonias? Dystonia is thought to be a circuit, network, or systems-level disorder, meaning that pathophysiology may involve abnormalities in any member of a functionally interdependent group of regions.[8, 9] Nevertheless, there may be shared predisposing factors across different types of dystonia, even if only some factors are shared between any two dystonias. We speculated in the current study that a more global method of structural assessment, direct measurement of regional brain volume, might identify more consistent structural abnormalities both within and across types of dystonia. Voxel-based studies require that abnormalities be colocalized across groups for detection. In contrast, volumetry captures a global assessment of each region, allowing for variation in exact location of the abnormality. Volumetric measures are also able to detect gross volume differences in the absence of changes in the cellular matrix structure.

While thalamic abnormalities (of diverse types) have been identified previously in dystonia, the substantial volumetric reduction observed in the current study offers additional insight into how thalamic dysfunction might contribute to some dystonias. For example, one might hypothesize (1) that widespread loss of inhibitory interneurons or afferent inhibitory axons would disinhibit thalamocortical projections, or (2) reduction of thalamic nuclei recognized to modulate thalamic afferents (e.g., the reticular nucleus) would decrease the precision of thalamic excitation, or (3) that with reduced volume of the thalamus comes a decreased ability to fine-tune and regulate motor program activity. Although the current study is vulnerable to many of the same limitations of any other single study, a compelling feature of the present study is our detection of patient/control differences that can be seen at the individual, as well as at the group, level. This suggests that our findings are not driven by an etiologic subgroup of patients (i.e., by the particular composition of our cohort).

While one might hypothesize that gross thalamic volume reductions observed in the current study reflect an increased rate of age-related thalamic atrophy in dystonia, there was no significant difference in age-related decline in our patients relative to controls, and even our youngest patients, and our patients with earliest onset, had notable reductions in thalamic volume (Fig 5A–5D). Furthermore, when comparing single patients to their own matched control (within a matched dyad), all but one of 17 dyads in the CD cohort, and all SD dyads showed patient < control for thalamic volume. This suggests that future studies can test the likelihood that all dystonia patients reliably fall outside the confidence interval for healthy subjects within a given age range. Furthermore, duration of symptoms did not correlate with thalamic volume (Fig 5E and 5F), arguing that thalamic volume loss was not driven by accumulated exposure to dystonia. In fact, for both CD and SD the lines of best fit for the relationship between duration and thalamic volume had positive, rather than negative slopes, suggesting this was not simply a sample size issue. For CD, we found no correlation between thalamic volume and either severity (Fig 5G) or laterality of BTX injections (Fig 5I), arguing that absolute volume differences were not related to asymmetries in clinical symptom severity or laterality. It is also important to emphasize that while there was a small improvement in the F statistic when age effects were removed, the increase in F value was minimal (from F = 19.385 to F = 21.348), suggesting that our tight matching and paired t-test evaluation across all regions counteracted any potential added variance with the broad age range in CD patients. However, given the modest size of the CD cohort, and the small size of the SD cohort, it is likely that determining clinical or demographic relationships in the data will require further work with larger cohorts to understand the clinical implications of our findings.

An alternate and more likely hypothesis is that reduced gross thalamic volume conveys susceptibility to these types of dystonia, but does not dictate the site of symptoms (cervical versus laryngeal). This lack of specificity stands in contrast with genetically based dystonias (such as mutations in *Tor1A* or *Thap1*), in which a predictable pattern of symptom site(s) is expected. However, it is important to note that we cannot rule out that the site of thalamic volume reduction (which nuclei or neuronal subpopulations) may influence the site of symptoms. Furthermore, while the presence of thalamic volume reduction alone does not appear to categorically dictate a precise age of symptom onset (e.g., onset range was 16 to 66 in the CD cohort), future studies are needed to determine whether the magnitude of volume reduction independently associates with age of onset. Similar to this hypothesis, cerebellothalamic tractography (a reflection of structural connectivity) is reduced in both disease-manifesting and non-manifesting carriers of dystonia-related mutations,[57] arguing that a developmental deficiency of thalamic connectivity predisposes to generalized dystonia, but may interact with additional factors that determine whether dystonia manifests.[57, 60, 61] Our findings also suggest that reduced thalamic volume may not relate to clinical variables in the same way in CD versus SD, although our sample sizes and demographic differences across samples did not allow us to test this in a rigorous manner. Future studies should be conducted in cohorts specifically designed to test these relationships.

It is important to note that subjects in the current study were scanned at a single time point, in a modest-sized cohort–it is unknown whether reductions in thalamic volume were longstanding and static or progressed with age, and to what extent such an effect would be observed across a substantially larger cohort. This underscores the need for future longitudinal and/or larger cohort studies to replicate and extend our findings, including determining whether any additional regions might emerge as significantly different from controls with greater statistical power. This is particularly true given the variable effect of head size on regional volumetry, although we corrected for this factor in the current study. Future efforts to identify the sources of reduced thalamic tractography and thalamic volume reduction (e.g., lack of a distributed neuronal population vs. reduction of an isolated thalamic nucleus vs. reduction in numbers of thalamic afferent/efferent axons) may help to elucidate the mechanisms by which a change in thalamic structure predisposes to dystonia.

Few studies have used manual anatomic segmentation to measure dystonia-related changes in gross, regional brain volume. Though manual volumetry is considered the “gold standard” for gross volumetric measurement, it is time consuming, requires experienced neuroanatomists, and is limited to *a priori* regions of interest. However, human experience and judgment are considered to be both more accurate and consistent than automated methods of segmentation.[62–66] Studies using manual volumetry in dystonia have successfully predicted clinical outcomes in a variety of regions and etiologies: predicting dystonia following thalamic stroke;[12] using gross volume of the globus pallidus to predict response to DBS;[11] segregating patients with Machado-Joseph disease into those with/without dystonia based on degree of thalamic atrophy.[13] Structural assessments of the basal ganglia and thalamus may be particularly difficult for automated segmentation techniques that utilize T1-weighted imaging. The higher iron content of deep grey structures and higher frequency of interstitial white matter bundles reduce the T1 contrast between grey and white matter, the basis for automated regional segmentation. Alternate imaging modalities, such as magnetization transfer imaging, may be better suited to automated segmentation of the basal ganglia and thalamus.[67]

The contrast of our thalamic segmentation findings with measures of VBM-volumetry in this region highlights the importance of using multimodal imaging to assess disorders,[18], especially those for which the underlying neurologic predisposition or pathology is unknown. None of the three VBM contrasts we used identified voxelwise volumetric differences in the thalamus. While both VBM and gross volumetry are tissue volume measures, gross volume evaluates the overall size of the brain structure (i.e., number of voxels) while VBM measures the tissue density within a given voxel and extrapolates a local volume measure from the Jacobian warp matrix. These measures are complementary, but measure different aspects of brain structure and should not be expected to change in lockstep. Each technique has limitations of resolution and specificity that may be balanced by the strengths of the other method. A decrease in gross volumetric measures argues strongly that something is missing or deficient, but cannot identify which cell populations or components are reduced. Similarly, local volume measures, through VBM, do not directly relate to underlying neuronal density or structure in a straightforward way: as noted by Zatorre et al., “Any tissue property that affects relaxation times (*e*.*g*., cell density, cell size, myelination), and hence affects voxel intensities on a T1-weighted image, will influence these measures.”[17] Recognizing the limitations of each method and therefore including multiple structural assessment techniques to study dystonia increases the likelihood of identifying the causes of this largely-idiopathic disorder.

Given that dystonia is thought to be a “network” or “systems level” disorder,[9, 24] and that both fMRI and VBM studies show considerable variability across forms of dystonia, it is not surprising that the field has yet to identify a “final common pathway” linking different phenotypes of the disorder. The gross thalamic volume change we identified, common across two forms of focal dystonia and persistent in the face of robust statistical correction, may be a clue to such a shared pathway. If other dystonias share this structural difference, future neuropathologic studies will be needed to (a) determine which cellular and/or axonal populations within the thalamus are altered (although our VBM results support the hypothesis that no one population is being affected in isolation), (b) evaluate whether this change arises from hypoplasia, remodeling, or atrophy, and (c) assess if this difference localizes to particular thalamic nuclei.

How might the assessment of gross thalamic volume be applied clinically, given replication with larger samples and the establishment of age-based confidence intervals? The clinical diagnosis of dystonia does not typically require imaging clarification. For patients with uncertain diagnoses (*e*.*g*., those with spasticity or rigidity and an overlying movement abnormality suggestive of dystonia, or when both an organic dystonia and a functional neurological disorder are considered), measurement of thalamic volume might solidify the diagnosis. In familial disorders without an identified genetic defect, thalamic volume could potentially predict future dystonia risk in unaffected relatives. Correlating thalamic volume with treatment efficacy (*e*.*g*., response to BTX or DBS) might allow prediction of benefit prior to undergoing invasive procedures. Such clinical uses are beyond the data in this series, and will require larger age- and gender-matched series to establish appropriate confidence intervals.

## Supporting Information

S1 Table Regional volume normalized to large-scale volumetric measures. Raw volume of each motor control region was normalized to either Freesurfer’s estimated intracranial volume (eTIV) or Freesurfer’s estimated whole-brain volume (BrainMaskVol) and expressed as volume relative to regional volume of matched controls (i.e., matched controls were set to 100% and patients were expressed as percentage of control volume). With both methods of normalization, only thalamic volume differed between patients and controls for both cervical dystonia and spasmodic dysphonia. Abbreviations: SEM, standard error of the mean; BA6, Brodmann Area 6.
(TIF)
