# Supplementary figures and images for: Thalamic Volume Is Reduced in Cervical and Laryngeal Dystonias
Source: PLoS One. 2016 May 12;11(5):e0155302. doi: 10.1371/journal.pone.0155302 (PMC4865047; doi:10.1371/journal.pone.0155302)

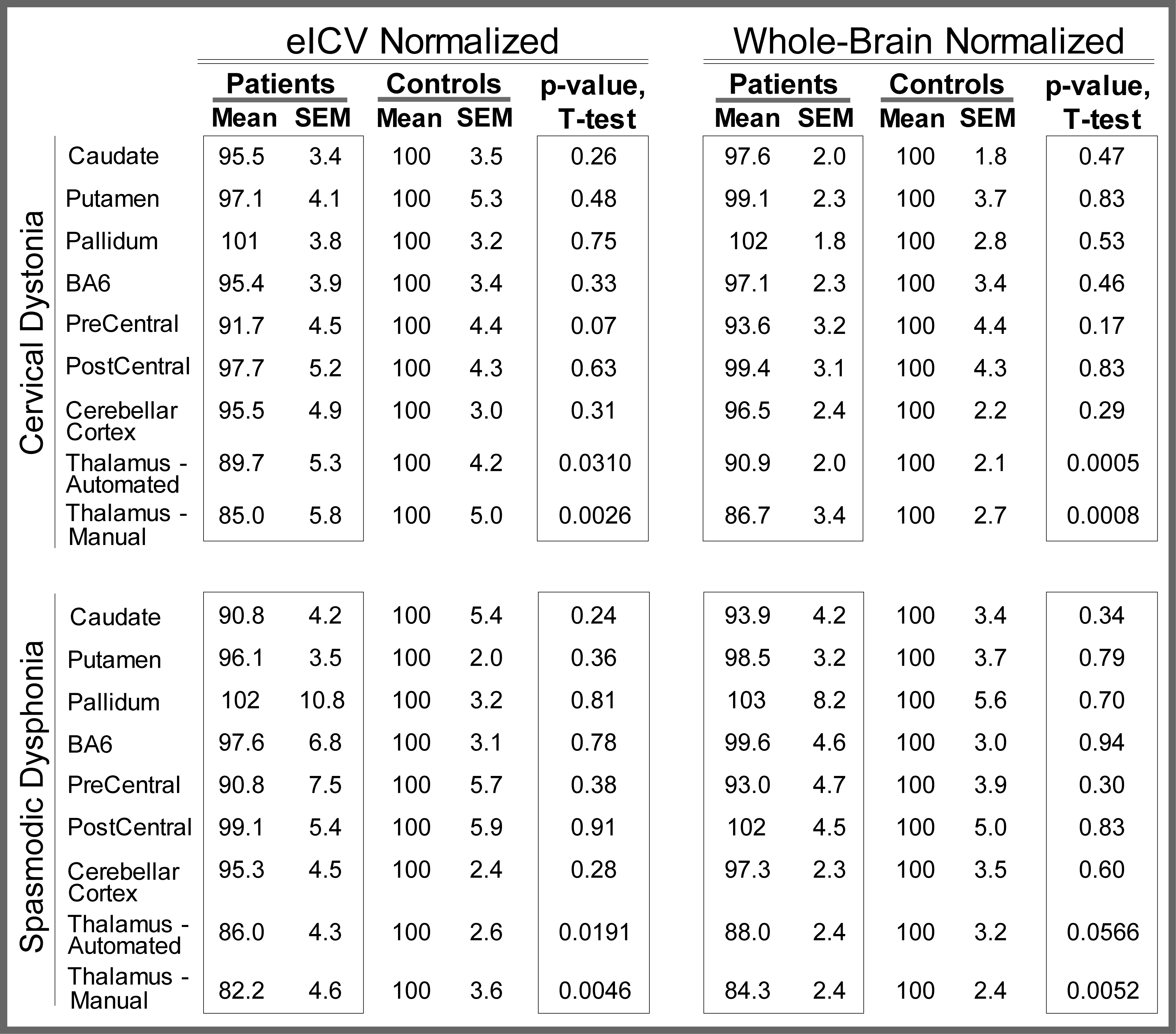

Supplement: S1 Table — Raw volume of each motor control region was normalized to either Freesurfer’s estimated intracranial volume (eTIV) or Freesurfer’s estimated whole-brain volume (BrainMaskVol) and expressed as volume relative to regional volume of matched controls (i.e., matched controls were set to 100% and patients were expressed as percentage of control volume). With both methods of normalization, only thalamic volume differed between patients and controls for both cervical dystonia and spasmodic dysphonia. Abbreviations: SEM, standard error of the mean; BA6, Brodmann Area 6. (TIF) [file pone.0155302.s001.tif]
